# Transcription factor EB agonists from natural products for treating human diseases with impaired autophagy-lysosome pathway

**DOI:** 10.1186/s13020-020-00402-1

**Published:** 2020-11-23

**Authors:** Jie Xu, Xiao-Qi Zhang, Zaijun Zhang

**Affiliations:** Guangdong Provincial Engineering Research Center for Modernization of TCM, Institute of New Drug Research, Guangdong Provincial Key Laboratory of Pharmacodynamic, Constituents of TCM and New Drug Research, College of Pharmacy, Jinan University, 510632 Guangzhou, People’s Republic of China

**Keywords:** TFEB, Autophagy, Autophagy-lysosome pathway, Natural products, TFEB agonists

## Abstract

Autophagy is a highly conserved degradation process for long-lived intracellular proteins and organelles mediated by lysosomes. Deficits in the autophagy-lysosome pathway (ALP) have been linked to a variety of human diseases, including neurodegenerative diseases, lysosomal storage disorders, and cancers. Transcription factor EB (TFEB) has been identified as a major regulator of autophagy and lysosomal biogenesis. Increasing evidence has demonstrated that TFEB activation can promote the clearance of toxic protein aggregates and regulate cellular metabolism. Traditional Chinese medicine (TCM)-derived natural products as important sources for drug discovery have been widely used for the treatment of various diseases associated with ALP dysfunction. Herein, we review (1) the regulation of TFEB and ALP; (2) TFEB and ALP dysregulation in human diseases; (3) TFEB activators from natural products and their potential uses.

## Background

There are two major protein degradation pathways in eukaryotic cells. The ubiquitin-proteasome system (UPS) is responsible for degrading short-lived soluble proteins, while the autophagy-lysosome pathway (ALP) is mainly responsible for regulating and recycling long-lived insoluble proteins and organelles. Autophagy is a lysosome-mediated bulk degradation process that occurs in all eukaryotic cells from yeasts to mammals [1]. Three main types of autophagy are currently recognized: macroautophagy, chaperone-mediated autophagy (CMA), and microautophagy. Although the mechanisms of the three subtypes are different, they have similar stimuli, such as environmental stress, nutrient starvation, oxidative stress, and infection [2].

In general, autophagy is divided into different stages, including initiation, elongation and maturation. Autophagy is initiated by the formation of a double-membrane structure called a phagophore. In the initiation of autophagy, there are two important autophagy initiation complexes. One is the UN51-like Ser/Thr kinases complex (ULK) [3]. Another essential autophagy complex for autophagosome formation is the class III phosphatidylinositol 3-kinase (PI3K) complex that is also called the beclin 1 complex [4, 5].

Then, two ubiquitin-like conjugation systems called the ATG12-ATG5-ATG16 complex and LC3/Atg8 are involved in the elongation of autophagy. The double-membrane phagophore elongates to engulf various intracellular cargos, including the damaged organelles and protein aggregates, and forms an autophagosomal vesicle. LC3-II is recognized as an autophagy marker [6]. Following the formation of the complete autophagosomal structure, the autophagosome fuses with the lysosome to form an autolysosome. Finally, in the autolysosome, the cargos are degraded by lysosomal enzymes to maintain cellular homeostasis.

In recent years, increasing numbers of studies have shown that deficits in the ALP are strongly associated with multiple diseases. Accordingly, correcting ALP defects and enhancing the activity of the pathway are promising therapeutic strategies.

Transcription factor EB (TFEB) was identified as a master regulator of autophagy and lysosomal biogenesis. TFEB binds to a promoter motif of the coordinated lysosomal expression and regulation (CLEAR) network, which consists of genes involved in processes such as autophagy, lysosomal biogenesis and membrane repair, and positively coordinates related downstream target genes [7]. It has been widely demonstrated that TFEB activators can ameliorate diseases related to ALP dysfunction, including neurodegenerative diseases, lysosomal storage disorders and so on [8, 9].

Owing to having fewer side effects and multitargeted mechanisms of action, traditional Chinese medicine (TCM)-derived natural products as important sources for drug discovery have great potential in the treatment of various ALP dysfunction-related diseases. In this review, we present an overview of the regulation of TFEB and ALP, the application of TFEB in ALP dysfunction-associated diseases, and TFEB activators derived from natural products. Furthermore, we look forward to identifying ideal TFEB activators with considerably higher specificity for the treatment of human diseases.

### The regulation of TFEB and ALP

#### The mTOR regulatory pathways

Mammalian (or Mechanistic) target of rapamycin (mTOR), a serine/threonine kinase, is the best-studied regulator of mammalian autophagy. Based on their structural differences, mTOR complexes are classified as mTOR complex (mTORC) 1 and mTORC2. mTORC1 is composed of mTOR, regulatory associated protein of mTOR (Raptor), G protein β-subunit-like protein (GβL), proline-rich Akt substrate of 40 kDa (PRAS40), and DEP domain-containing mTOR-interacting protein (DEPTOR) [2], and it responds to multiple stresses, including nutrients, growth factors and cellular energy status [10].

Under nutrient-rich conditions, PRAS40 is phosphorylated by Akt and dissociates from Raptor to activate mTORC1. Activated mTORC1 phosphorylates ULK1 at Ser757 to inhibit the ULK1 activity involved in autophagosome formation. However, AMP-activated protein kinase (AMPK) phosphorylates ULK1 at the Ser317 and Ser777 sites to promote ULK1 activity and autophagy. Although mTORC1 is the major sensor of nutrition and growth factor signals, autophagy can also be regulated by mTORC2 through the mTORC2-Akt-FoxO3 signaling pathway [11].

mTORC2 is composed of mTOR, rapamycin-insensitive companion of mTOR (Rictor), GβL, stress-activated protein kinase-interacting protein (SIN) 1, and protein observed with Rictor (PROTOR) [2]. mTORC2 can participate in autophagy regulation through the FOXO3 pathway. FoxO3 is a transcription factor that is activated under starvation conditions and promotes the transcription of genes that regulate autophagy induction [12]. mTORC2 phosphorylates Akt at Ser473, followed by Akt phosphorylation of FOXO3. Phosphorylated FOXO3 binds to the 14-3-3 protein, which retains it in the cytoplasm, preventing activation of autophagy gene transcription.

#### The regulation of TFEB

**Fig. 1:**
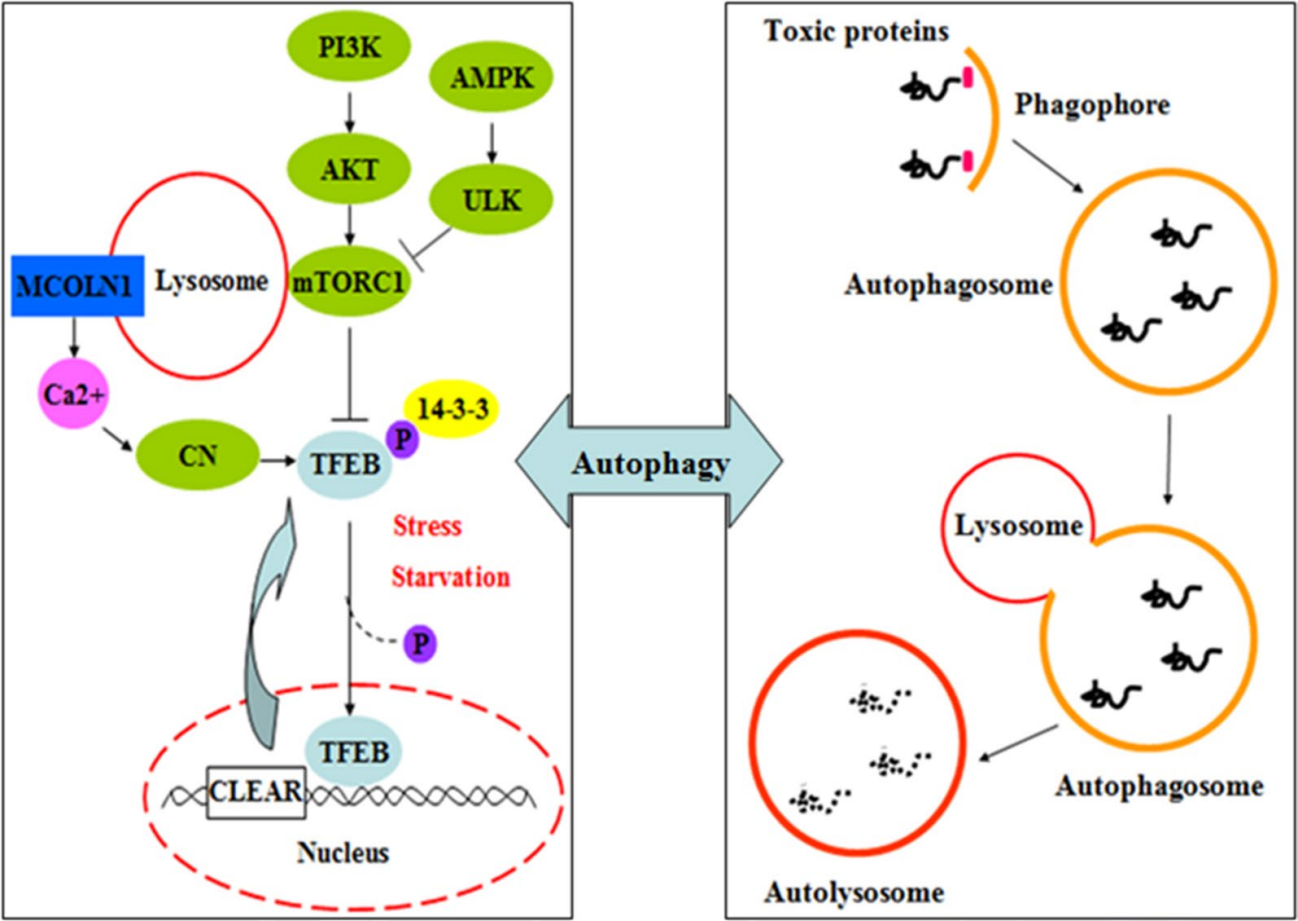
The regulation of TFEB and ALP. mTORC1 holds TFEB in a phosphorylated state. Phosphorylated TFEB binds to the 14-3-3 protein, which sequesters it in the cytoplasm. However, mTORC1 dependent/independent pathways that activate TFEB can dephosphorylate TFEB, and dephosphorylated TFEB translocates to the nucleus and binds to the CLEAR sequence, leading to upregulation of the autophagy and lysosomal genes. *PI3K* class III phosphatidylinositol 3-kinase, *AMPK* AMP-activated protein kinase, *ULK* UN51-like Ser/Thr kinases complex, *mTOR* Mammalian (or Mechanistic) target of rapamycin, *mTORC* mTOR complex, *CLEAR* coordinated lysosomal expression and regulation, *TFEB* transcription factor EB, *MCOLN1* calcium channel mucolipin 1, *CN* calcineurin

TFEB is a basic helix-loop-helix leucine zipper transcription factor, a member of the MiT family, that regulates metabolism and cellular clearance as a master regulator of the ALP (Fig. 1) [13]. mTORC1 and extracellular signal-regulated kinase 2 (ERK2, also known as MAPK1) are the two main kinases known to phosphorylate TFEB under nutrient-rich conditions in most cell types [14]. Under normal conditions, such as nutrient availability and no lysosomal stress, mTORC1 phosphorylates TFEB at the Ser142 and Ser211 sites. Phosphorylated TFEB binds to 14-3-3 protein, which sequesters it in the cytoplasm. However, under conditions of starvation or lysosomal stress, mTORC1 is released from the lysosomal membrane and becomes inactive, and no longer phosphorylates TFEB. The unphosphorylated TFEB translocates into the nucleus, where it promotes the transcription of its target genes [15, 16]. TEEB is identified to bind to the CLEAR sequence, leading to upregulation of the autophagy and lysosomal genes. The expression of autophagic and lysosomal target genes, such as Tfeb, Atg9b, and Sqstm 1, etc., can be positively regulated by TFEB [15].

The TFEB promoter contains multiple CLEAR sequences, and thus TFEB upregulates its own expression in an autoregulatory loop. Both the fed-state sensing nuclear receptor farnesoid X receptor (FXR) and the fasting transcriptional activator cAMP response element-binding protein (CREB) regulate the expression of TFEB. CREB up-regulate TFEB expression by recruiting the coactivator CRTC2. However, FXR trans-repress autophagy genes by disrupting the functional CREB–CRTC2 complex [17]. In addition, many factors regulate TFEB activity. The peroxisome proliferator-activated receptor-γ coactivator 1α (PGC-1α, also known as PPARGC1A) has been identified as a direct TFEB target [18]. The activation of nuclear receptor peroxisome proliferator-activated receptor α (PPARα) leads to nuclear translocation of TFEB and induces TFEB expression [19]. At the same time, PPARα, retinoid X receptor α (RXRα), and PGC-1α form a transcriptionally active complex. And the activation of PPARα can induce the recruitment of the PPARα-RXRα-PGC-1α complex on the TFEB promoter, thus regulating lysosomal biogenesis [20].

TFEB is regulated by another positive feedback loop. The calcium channel mucolipin 1 (MCOLN1), a transcriptional target of TFEB, creates a microdomain of high Ca^2+^ concentration near the lysosomal membrane upon nutrient deprivation [21]. The higher Ca^2+^ concentration leads to the activation of phosphatase calcineurin, which dephosphorylates TFEB, promoting TFEB nuclear translocation and the transcription of target genes [21]. Knockdown of the Rag GTPases, a part of the lysosomal nutrient-sensing machinery that signals to mTORC1, induces the nuclear translocation of TFEB even in nutrient-rich conditions [22]. The mutation of serine to alanine on TFEB abolishes its phosphorylation, and it then shows a significantly increased nuclear localization [16, 23]. More recently, endoplasmic reticulum stress has been identified to induce the nuclear translocation of TFEB as part of the integrated stress response. In this case, TFEB is activated by an RNA-like endoplasmic reticulum kinase (PERK/EIF2AK3)-dependent mechanism that promotes activation of calcineurin and nuclear translocation of TFEB [24].

### ALP and TFEB dysregulation-associated human diseases

#### Lysosomal storage disorders (LSDs)

LSDs are caused by the accumulation of undegraded materials in the lysosomal lumen due to a genetic deficiency in specific lysosomal proteins. The clinical outcome of undigested matrix storage in multiple organs and systems leads to variable neurological, visceral and skeletal manifestations [25].

Pompe disease as a severe metabolic myopathy is caused by acid alpha-glucosidase (GAA) deficiency, which is an enzyme responsible for breaking down glycogen to glucose, leading to an accumulation of glycogen. Pompe disease is characterized by both lysosomal abnormalities and autophagic dysfunction. Overexpression of TFEB in cell systems and a mouse model of this disease decreases glycogen accumulation and lysosomal size, improves autophagosome processing, and relieves the excessive load of autophagic vacuoles [26].

It has been reported that TFEB activation enhances the folding, trafficking and lysosomal activity of destabilized glucocerebrosidase (GC) mutants, which are associated with the development of Gaucher disease. Moreover, TFEB activation also rescues the activity of the β-hexosaminidase mutant involved in the development of another LSDs, Tay-Sachs disease [27]. Multiple sulfatase deficiency (MSD), a severe type of LSDs, is caused by defective posttranslational activation of sulfatase-modifying factor 1 and simultaneous deficiency of all sulfatases, leading to accumulation of glysosaminoglycans (GAGs) and aberrant autophagy.

Another LSD, known as mucopolysaccharidosis (MPS) type IIIA, which is caused by heparan sulfamidase deficiency, results in the progressive accumulation of GAGs and cellular vacuolization. TFEB overexpression in glia-differentiated neuronal stem cells (NSCs) derived from mouse models of MSD and MPS-IIIA significantly reduces the level of intracellular GAGs, decreases cellular vacuolization, and restores normal cell morphology. Notably, TFEB overexpression rescues not only the GAGs accumulation but also secondary pathological processes associated with LSDs such as inflammation and cell death seen in vivo in MSD [28].

Overexpression of TFEB reduces the accumulation of lipofuscin in cell models derived from mice and patients with Batten disease, which is caused by mutations of the CLN3 gene [28]. Cystinosis is an autosomal recessive LSDs characterized by the accumulation of cystine into lysosomes. Cystinosis is caused by mutations in the CTNS gene, which encodes the cystine lysosomal transporter, cystinosin. The deficiency or dysfunction of cystinosin causes the accumulation of cystine in lysosomes throughout the body. Lack of cystinosin decreases the TFEB level and induces TFEB nuclear translocation. Both genetic and chemical activation of TFEB are able to reduce cystine stores, stimulate delayed cargo processing, and rescue the aberrant lysosomal compartment morphology in cystinotic cells [29].

The mechanism of TFEB-mediated intracellular clearance not only includes the enhancement of lysosomal activity and biogenesis but also TFEB-induced lysosomal exocytosis. TFEB-induced lysosomal exocytosis requires lysosomal recruitment to the cell surface in a Ca^2+^-independent manner first and TFEB enhances Ca^2+^-mediated fusion of lysosomes with the plasma membrane via activation of the lysosomal Ca^2+^ channel MCOLN1 [28]. In summary, TFEB-mediated proteostasis regulation generally rescues destabilized mutations in LSDs, and this suggests that TFEB may be an appealing target to rescue enzyme homeostasis in LSDs.

#### Neurodegenerative diseases

Neurodegenerative diseases, which are age-dependent diseases caused by the loss of neurons and spinal marrows, leading to functional disorders of the brain, are characterized by intracellular accumulation of aggregate-prone proteins and damaged protein degradation systems. As is widely acknowledged, the ALP pathway in neurodegenerative diseases plays an essential role in the clearance of intracellular misfolded protein aggregates to maintain homeostasis.

Huntington’s disease (HD) is caused by trinucleotide CAG repeat expansions in the first exon of the huntingtin (HTT) gene. The CAG repeat is translated into an expanded polyglutamine (polyQ) tract in the amino terminal region of the HTT protein, resulting in misfolding into a pathogenic conformation. Recently, overexpression of TFEB was found to reduce intracellular HTT protein aggregation in a mouse model of HD. In addition, TFEB has been identified as a downstream transcriptional target of PGC-1α, which has been shown to ameliorate the symptoms of HD mice through activation of TFEB [30].

Parkinson’s disease (PD) is a progressive neurodegenerative disease affecting dopaminergic neurons in the substantia nigra. PD is pathologically characterized by the accumulation of proteinaceous cytoplasmic inclusions termed Lewy bodies, containing misfolded and aggregated α-synuclein [31]. Furthermore, PD is accompanied by lysosomal deficiency. Overexpressing TFEB or inducing its nuclear translocation eliminates this deficit and attenuates α-synuclein pathology [8, 32]. Another pathological hallmark of PD is the accumulation of damaged mitochondria due to mitophagy dysfunction. It has been reported that the PINK1-Parkin pathway plays an essential role in regulating the selective elimination of damaged mitochondria via mitophagy. TFEB translocates to the nucleus and displays transcriptional activity in a PINK1-Parkin-dependent manner [33]. In addition, Parkin, through its effects on PARIS, plays a crucial role in overall mitochondrial homeostasis via cellular regulation of the PGC-1α-TFEB signaling pathway [34].

Alzheimer’s disease (AD) is the most common form of neurodegenerative disease in the aged. Extracellular amyloid plaques consisting of β-amyloid peptides (Aβ) and intracellular neurofibrillary tangles (NFTs) composed of hyperphosphorylated tau protein are considered the main pathological characteristics of AD. AD is correlated with genetic origins, such as mutations in amyloid precursor protein (APP) and presenilin (PS) 1 and 2. [35]. The impaired clearance of these aggregation-prone proteins results in neurotoxicity, neurodegeneration, and memory deficits. In addition to the UPS and CMA pathways, the selective autophagic clearance of aggresomes, termed aggrephagy, is activated. Accumulating evidence has demonstrated that TFEB can attenuate protein aggregates in cell and mouse models of AD and other tauopathies, resulting in alleviation of neurodegeneration and improvement of behavioral deficits, as well as the recovery of cognitive impairment [9, 36].

Spinal and bulbar muscular atrophy (SBMA), known as Kennedy disease, is a motor neuron disease. It is caused by CAG repeat expansion in the first exon of the androgen receptor (AR) gene. According to several studies, autophagy is involved in the pathogenesis of SBMA. The expression of TFEB can activate autophagy, reduce aggregation of the abnormal AR protein, and alleviate the motor phenotypes [37].

Amyotrophic lateral sclerosis (ALS) is characterized by the formation of protein inclusions and the degeneration of motor neurons. ALS pathogenesis is associated with several mutations. Notably, the transactive response (TAR) DNA-binding protein 43 kDa (TDP-43) aggregate has been identified as the major component of protein inclusions in ALS. The activation of TFEB enhances autophagy and the clearance of TDP-43 aggregates [38].

In conclusion, promoting the function of ALP by activating TFEB may be a therapeutic strategy for neurodegenerative disorders.

#### Cancers

Cancer is characterized by a genetic and metabolic imbalance causing abnormal cell growth. Autophagy, serving as a double-edged sword due to its complex effects on cancer, displays both pro-tumorigenic and anti-tumorigenic effects [39, 40].

Autophagy is a catabolic process used to degrade long-lived proteins and cytoplasmic components, and then it supports tumor progression through reusing the produced metabolites to synthesize new macromolecules or using them as an energy supply. Glycogen synthase kinase-3 (GSK3) inhibition triggers pro-survival signals by increasing the activity of the autophagic/lysosomal network. TFEB overactivation increases the proliferation of pancreatic cancer cells [41]. Chromosomal translocations involving TFEB and TFE3 are found in renal cell carcinoma [42]. TMEM106B modulates the expression of the CLEAR network lysosomal genes in lung cancer cells in a TFEB-dependent manner, and drives lung cancer metastasis [43]. Moreover, autophagy can also protect against tumors by depriving them of nutrients and restricting cell proliferation. Hence, the role of autophagy in tumorigenesis is controversial because of the crosstalk between autophagy and apoptosis.

#### Other diseases

It has been documented that TFEB can control lipid catabolism by regulating PGC-1α, a key regulator of lipid metabolism. TFEB overexpression in the liver prevents weight gain and associated metabolic syndrome in both diet-induced and genetic mouse models of obesity [44]. TFEB, as a PGC-1α-dependent regulator of adipocyte browning, has therapeutic potential in metabolic dysfunction. Adipocyte-specific TFEB overexpression in mice is protective against diet-induced obesity, insulin resistance, and metabolic sequelae [18].

Age-associated cardiovascular diseases are characterized by increased oxidative stress associated with autophagy dysfunction. Reactive oxygen species (ROS) can not only impair ventricular function but also block autophagy [45, 46]. Monoamine oxidase (MAO) is a potent ROS source in several cardiomyopathies. MAO-A can degrade catecholamine and serotonin to produce hydrogen peroxide (H_2_O_2_), which results in oxidative stress. TFEB overexpression attenuates the negative impacts of the MAO-A/H_2_O_2_ axis by reducing autophagosome accumulation and cardiomyocyte death [47]. Furthermore, the nuclear translocation and activation of TFEB can induce autophagy and confer cardioprotection in cardiomyocytes with overexpression of MAO-A [48]. Atherosclerosis is the most serious threat to human cardiovascular health. Endothelial oxidative injury is a driving force in the pathogenesis of atherosclerosis. In human umbilical vein endothelial cells (HUVECs), endothelial oxidative injury stimulated by palmitic acid is counteracted by inducing autophagy in a TFEB-dependent manner [49].

TFEB gene transfer is available for the treatment of liver disease caused by alpha-1-anti-trypsin deficiency [50]. Recent evidence has suggested that TFEB has a broad effect on modulating inflammatory reactions, immune responses [51, 52] and bone resorption [53].

### TFEB activators from natural products

**Fig. 2:**
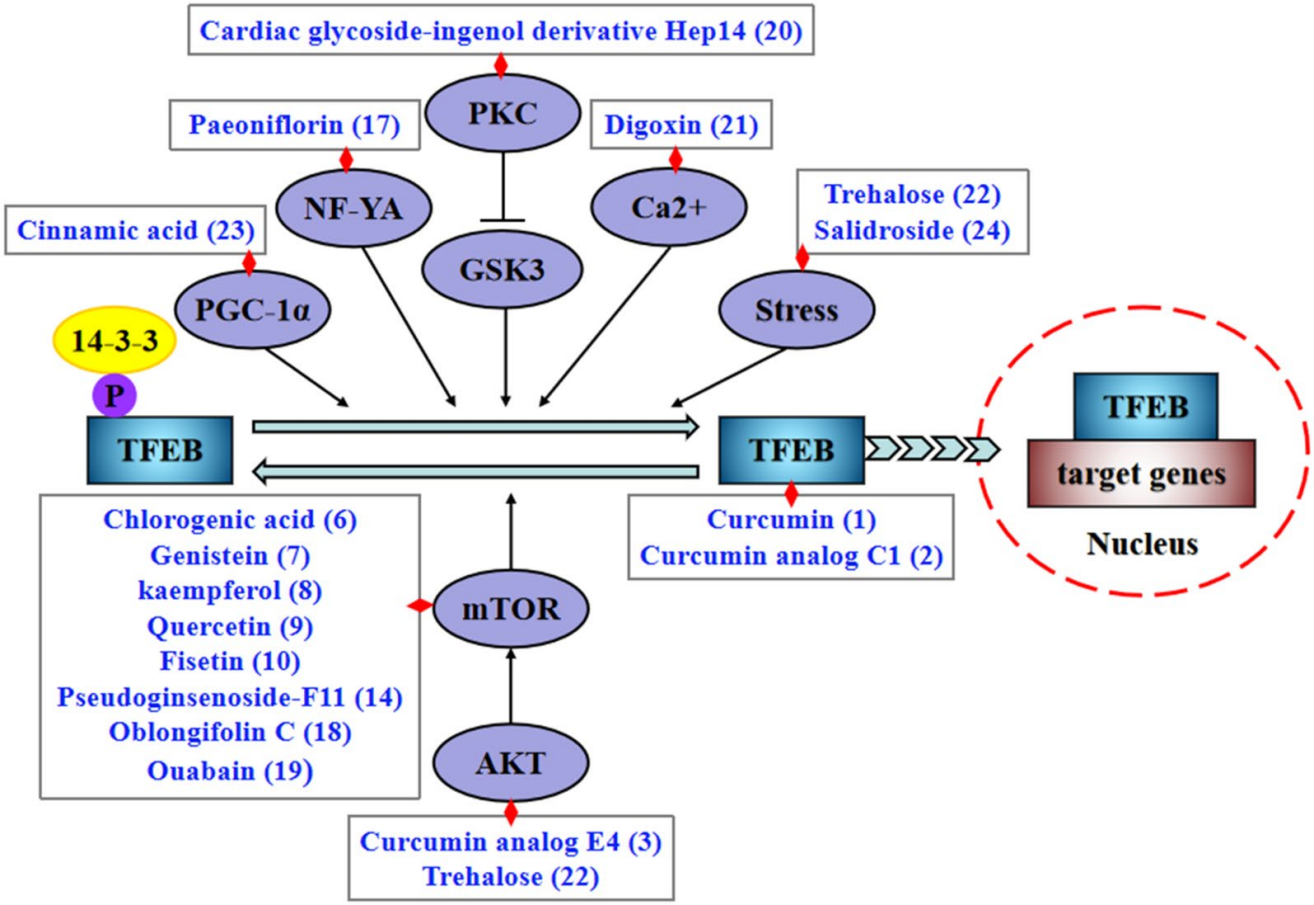
The TFEB activators from natural products. Phosphorylated TFEB binds to the 14-3-3 protein, which sequesters it in the cytoplasm. Some active ingredients of TCM can dephosphorylate TFEB and translocate TFEB to the nucleus via multiple mechanisms. *PKC* protein kinase C, *TFEB* transcription factor EB, *mTOR* Mammalian (or Mechanistic) target of rapamycin, *PGC-1α* peroxisome proliferator-activated receptor-γ coactivator 1α, *NF-YA* nuclear factor-YA

TFEB, a master regulator of autophagy and lysosomal biogenesis, has become an attractive target for alleviating ALP dysfunction. Owing to their lesser side effects and multitargeted action, TCM-derived natural products have therapeutic promise for multiple diseases. Some active ingredients from TCM have been reported to activate TFEB via multiple mechanisms, including mTOR inhibition, Akt inhibition, and Ca^2+^-dependence, as well as direct TFEB activation and so on (Fig. 2). Additionally, these active ingredients can regulate autophagic and lysosomal function, enhance the clearance of toxic aggregates, engage in apoptosis by activating TFEB (Table 1).

**Table 1:** Pharmacological effect of TFEB activators for the treatment of ALP dysfunction

Compound	Disease	Mechanism involved in activation of TFEB	Model	Effects	References
Curcumin (**1**)	Cancer	Directly bind to TFEB	Human colon cancer HCT116 cells and MEFs	Enhance autophagic flux, promote lysosomal function	[54]
Curcumin analog C1 (**2**)	1. AD 2. PD	Directly bind to TFEB	1. 5×FAD mice, P301S mice, 3xTg-AD mice 2. SH-SY5Y cells, iPSC-derived DA neurons, mice nigral DA neurons, 6-OHDA/AA-lesioned models	1. Reduce APP, CTF-β/α, Aβ and Tau aggregates, improve motor and cognitive function 2. Enhance autophagy, reduce neuronal death, rescue behavioral abnormality	[55, 56]
Curcumin analog E4 (**3**)	PD	Inhibit Akt-mTORC1	N2a cells, HeLa cells, PC12 cells	Promote autophagic flux and lysosomal biogenesis, reduce α-synuclein, protect against MPTP toxicity	[57]
Resveratrol (**4**)	Atherosclerosis		HUVECs	Promote autophagic flux, attenuate endothelial oxidative injury	[49]
Oleuropein aglycone (**5**)	Cardiovascular disease		Cardiomyocytes	Promote autophagic flux, restore autophagy impairment, protect from cardiotoxicity	[48]
Chlorogenic acid (**6**)	AD	Inhibit mTOR	Aβ25-35-induced SH-SY5Y cells APP/PS1 mice	Ameliorate cognitive deficits, neuronal injury, and Aβ plaque deposition, inhibit The production of autophagosomes, improve autophagic flux, enhance lysosomal activity	[58]
Genistein (**7**)	MPS		HDFa cells, HeLa cells, MEFs	Inhibit GAG synthesis, enhance lysosomal hydrolases	[59]
Genistein (**7**) and kaempferol (**8**)	MPS	Inhibit mTOR	HDFa and MPS II cells	Stimulate the expression of genes coding for GAG degrading enzymes	[60]
Quercetin (**9**)	Neuronal tissues	Inhibit mTORC1	Retinal pigment epithelium cells	Enhance autophagy, degrade phagocytosed photoreceptor outer segments	[61]
Fisetin (**10**)	AD	Inhibit mTOR	Mouse cortical neuronal cells, rat primary cortical cells, HEK293 cells	Enhance autophagy, degrade p-tau and sarkosyl insoluble tau	[62]
Corynoxine isomers (**11–12**)	AD		N2a cells, Tg2567 mice	Decrease Aβ, APP and CTF, promote autophagy and lysosome biogenesis	[63]
Fangchinoline (**13**)	Cancers		NSCLC cells	Inhibit the fusion of autophagosome and lysosome, decrease autophagic flux, affect lysosome function	[64]
Pseudoginsenoside-F11 (**14**)	AD	Inhibit mTOR	Microglial cells	Increase uptake and degradation of oligomeric Aβ, promote maturation of endosome, improve lysosomal function	[65]
Gypenoside XVII (**15**)	AD		PC12 cells expressing the Swedish mutant of APP695, APP/PS1 mice	Improve autophagic flux, enhance lysosome biogenesis, degrade Aβ, restore the spatial learning and memory	[66]
Hinokitiol (**16**)	Cancers		NSCLC cells, HCC827 cells, HeLa cells, MEFs	Promote autophagy and lysosomal biogenesis, induce cancer cells death	[67]
Paeoniflorin (**17**)	SBMA	Increase the expression of NF-YA to upregulate TFEB	NSC34 cells stably expressing AR-97Q, AR-97Q mice	Activate autophagy, inhibit aggregation of the mutant AR protein, ameliorate motor phenotypes	[37]
Oblongifolin C (**18**)	Cancers	Inhibit mTORC1	HeLa cells, L929 cells, MEF cells, HEK293T cells	Improve autophagosome maturation, block autophagosome–lysosome fusion, suppress HeLa cells growth	[68]
Ouabain (**19**)	AD	Inhibit mTOR and Na^+^/K^+^-ATPase	HeLa and SH-SY5Y cells, primary cortical neurons, drosophila melanogaster tau model, Tau-P301L mice	Relieve neuronal damage, reduce p-Tau aggregates, improve a rough-eye phenotype, restore memory performance	[69]
Cardiac glycoside-ingenol derivative Hep14 (**20**)	AD	Activate PKC, PKC inactivate GSK3 leading to TFEB dephosphorylation	HeLa and HepG2 cells, APP/PS1 mice	Activate lysosomal gene expression, promote lysosome-dependent clearance of lipid droplets and aggregated proteins	[70]
Digoxin (**21**)	Metabolic disorders	Ca^2+^-dependent TFEB dephosphorylation to activate TFEB	HepG2 Cells, C. *elegans*, C57BL/6 J mice	Ameliorate metabolic syndromes, extend lifespan	[71]
Trehalose (**22**)	1. ALS 2. ALS, SBMA 3. Batten disease	1. mTOR-independence 2. PPP3B mediated TFEB dephosphorylation and lysosomal stress 3. inhibit Akt	1.SH-SY5Y and Hella cells 2. NSC34 cells 3. Cln3Dex7-8 mice	1. Enhance autophagy flux, eliminate TDP-43 aggregates 2. Degrade misfolded protein including AR. Q46, TDP-43, and mutant SOD1 3. Enhance cellular clearance of proteolipid aggregates, reduce neuropathology	[38, 72, 73]
Cinnamic acid (**23**)	AD	Activate PGC-1α to transcriptionally upregulate TFEB expression	Mouse primary brain cells, 5xFAD mice	Induce lysosomal biogenesis, reduce the amyloid plaque burden, improve memory and behavioral performance	[74]
Salidroside (**24**)	Cancers	Induce ROS generation, ROS activate the TFEB signal pathway	SW1353 cells	Induce autophagy, promote apoptosis	[75]

‘–’ indicates absence of data

### Polyphenols

#### Curcumin and its analogs

Curcumin (**1**, Fig. 2) is a hydrophobic polyphenol isolated from *Curcuma longa* L., exhibiting diverse pharmacologic effects [76–78]. It has been extensively demonstrated that curcumin induces autophagy by suppressing the PI3K-Akt-mTOR signaling pathway or directly activating TFEB. It has been documented that curcumin directly binds to TFEB, promotes TFEB nuclear translocation, reduces the phosphorylation level of TFEB, and increases the transcriptional activity of TFEB. Moreover, curcumin enhances autophagic flux in human colon cancer HCT116 cells and mouse embryonic fibroblasts (MEFs), and promotes lysosomal function via suppression of mTOR. Curcumin-induced lysosomal activation promotes cell survival, and lysosome inhibition is able to cause more cell death in curcumin-treated HCT116 cells, which may facilitate the development of curcumin as an anticancer agent [54].

However, due to the poor absorption and low bioavailability of curcumin, several derivatives of curcumin have been chemically synthesized to improve its bioavailability and potency. A synthesized curcumin monocarbonyl derivative termed C1 (**2**, Fig. 2) has been identified as a potent mTOR-independent activator of TFEB. Compound C1 directly binds to TFEB at the N terminus and promotes TFEB entry into the nucleus, without affecting TFEB phosphorylation or inhibiting the activities of mTOR and MAPK1/ERK2-MAPK3/ERK1. C1 is effective in enhancing autophagy and lysosome biogenesis in vitro and in vivo [79]. C1, an mTOR-independent activator of TFEB, efficiently reduces APP, APP C-terminal fragments (CTF-β/α), Aβ and Tau aggregates in three AD animal models, such as beta-amyloid precursor protein pathology (5xFAD mice), tauopathy (P301S mice) and the APP/Tau combined pathology (3xTg-AD mice). At the same time, C1 improves the motor and cognitive function of mice models [55]. C1 enhances TFEB nuclear translocation and autophagy in 6-hydroxydopamine/ascorbic acid (6-OHDA/AA)-lesioned models of PD to exert neuroprotective effects. C1 significantly reduces neuronal death in SH-SY5Y cells, iPSC-derived DA neurons and mice nigral DA neurons, and rescues the behavioral abnormalities of 6-OHDA/AA treated mice [56]. Furthermore, C1 promotes the transport of Hex and Gal from lysosomes to the plasma membrane via mTORC1-independent TFEB activation [80]. Curcumin monocarbonyl analog E4 (**3**, Fig. 2) induces TFEB activation through Akt-mTORC1 inhibition. E4 promotes autophagy flux and lysosomal biogenesis, reduces α-synuclein levels and protects against 1-methyl-4-phenyl-1,2,3,6-tetrahydropyridine (MPTP) toxicity in vitro [57].

#### Resveratrol

Resveratrol (**4**, Fig. 2) is a natural polyphenol that is mainly isolated from grapes. Resveratrol is associated with multiple health benefits, such as neuroprotective and anti-atherosclerosis effects. Resveratrol activates TFEB in HUVECs, and ameliorates endothelial oxidative injury by inducing autophagy [49]. In addition, TFEB can be acetylated, inhibiting gene transcription, while SIRT1 can deacetylate TFEB and improve the transcription of its target genes [81]. Resveratrol, as a SIRT1 activator, can promote the nuclear translocation of TFEB and upregulate the target gene levels of TFEB [82].

#### Oleuropein aglycone

Oleuropein aglycone (**5**, Fig. 2), the main polyphenol found in olive oil, has been proven to activate autophagy against neurodegeneration [83, 84]. Oleuropein aglycone can translocate TFEB to the nucleus and upregulate TFEB target genes. Oleuropein aglycone regulates autophagic flux in cardiomyocytes and restores autophagy impairment resulting from MAO-A induced oxidative stress, protecting against cardiotoxicity [48].

#### Chlorogenic acid

**Fig. 3:**
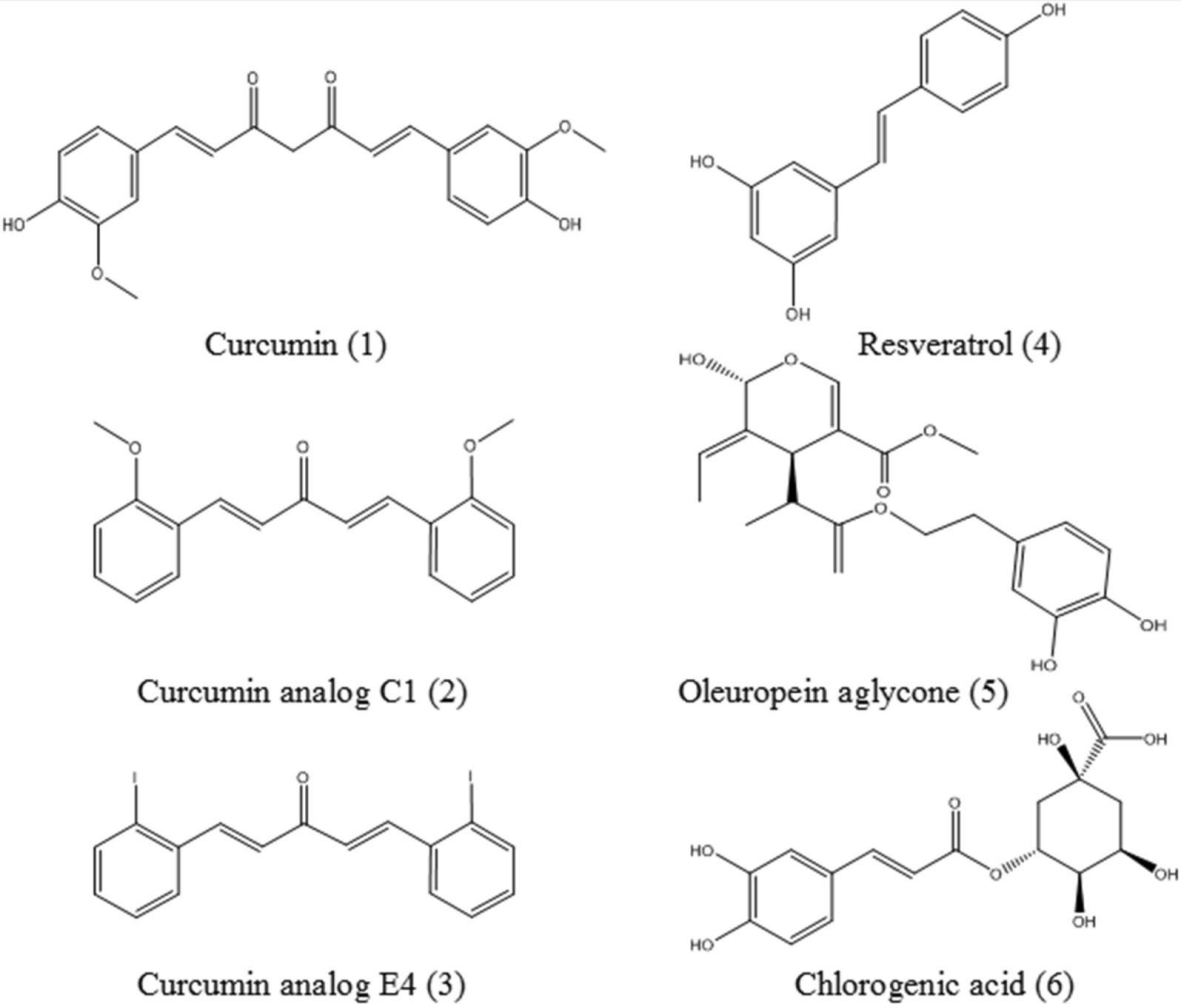
Chemical structures of polyphenols, including curcumin, curcumin analog C1, curcumin analog E4, resveratrol, oleuropein aglycone and chlorogenic acid

Chlorogenic acid (**6**, Fig. 3) is a phenolic acid compound extracted from honeysuckle, tea and coffee [85, 86]. Chlorogenic acid promotes TFEB nuclear translocation and increases TFEB protein levels by the mTOR-TFEB signaling pathway. Chlorogenic acid can inhibit the production of autophagosomes, improve the fusion of autophagosomes with lysosomes, and enhance lysosomal function in vitro and in vivo. Moreover, chlorogenic acid effectively ameliorates cognitive deficits, neuronal injury, and Aβ plaque deposition in APP/PS1 mice [58].

### Flavonoids

#### Genistein and kaempferol

**Fig. 4:**
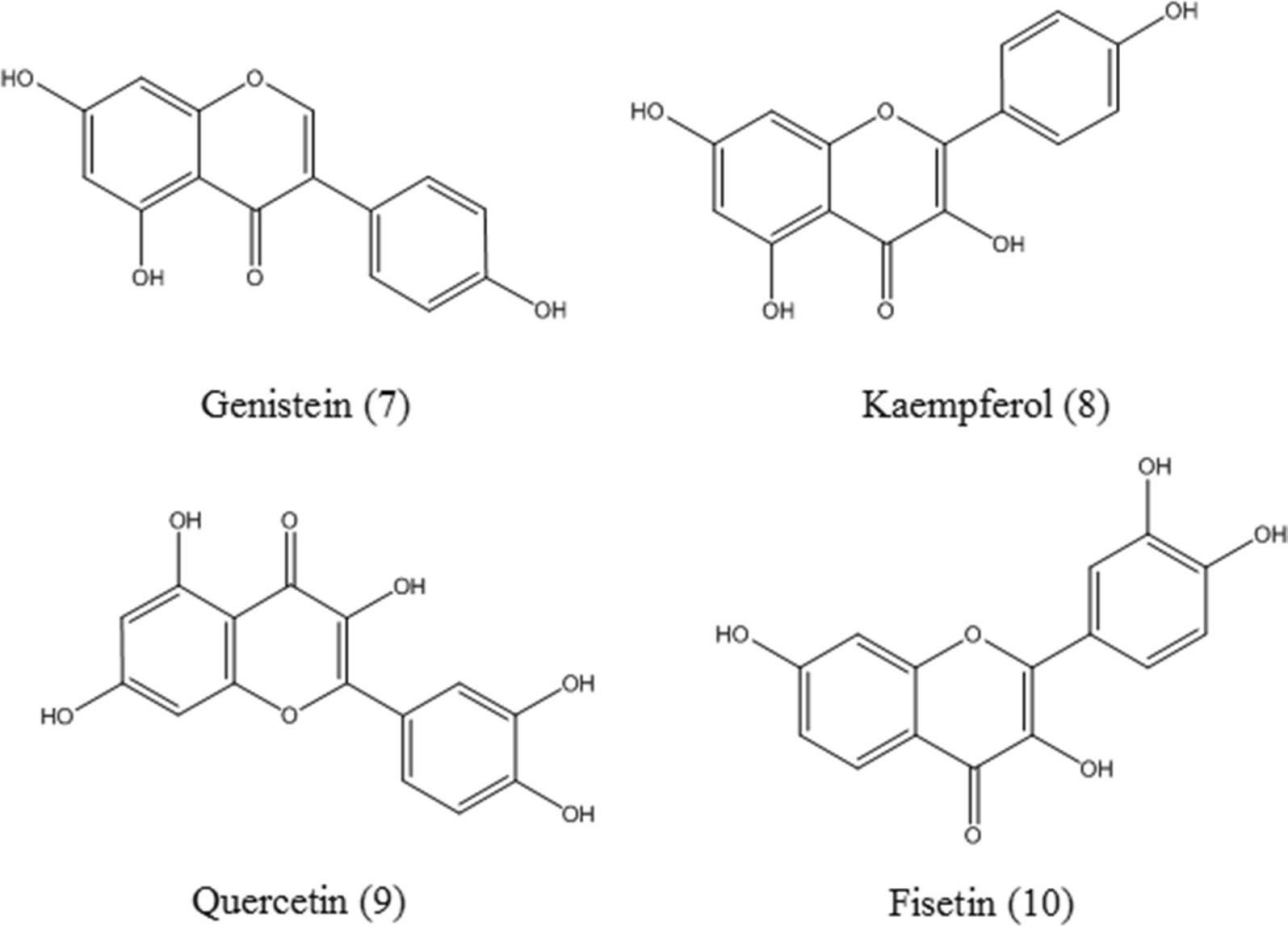
Chemical structures of flavonoids, including genistein, kaempferol, quercetin, and fisetin

Genistein and kaempferol (**7**–**8**, Fig. 4) are natural flavonoids. Genistein is able to activate TFEB and increase mRNA levels of representative CLEAR network genes, such as SQSTM1, CTSD, SMPD1, and reduce intracellular cystine levels in cystinotic cells [29]. In addition, genistein promotes the nuclear translocation and target gene levels of TFEB. Genistein not only inhibits the expression of genes involving in GAG synthesis but also enhances the expression of genes coding for various lysosomal hydrolases [59]. It has been observed that genistein, kaempferol and a mixture of them can increase TFEB expression and decrease the levels of mTOR transcripts in both HDFa and MPS II cells, which may stimulate the expression of genes coding for GAG degrading enzymes [60]. Therefore, it offers therapeutic strategies for the treatment of some LSDs.

#### Quercetin

Quercetin (**9**, Fig. 4) is a plant-derived flavonoid compound. Quercetin treatment activates TFEB by inhibiting mTORC1 in the retinal pigment epithelium cells. Activated TFEB facilitates the degradation of phagocytosed photoreceptor outer segments [61]. However, the concentration of quercetin to effectively activate TFEB is relatively higher than the reported blood concentration seen in human clinical experiments. Therefore, studies of its pharmacokinetic and pharmacodynamic properties are necessary; And pharmaceutical preparation approaches to improve the bioavailability of quercetin is needed.

#### Fisetin

Fisetin (**10**, Fig. 4) is a flavonol from *Rhus succedanea* L. Fisetin has been recently reported to activate TFEB via mTOR inhibition and stimulate autophagic degradation of phosphorylated tau in neurons [62].

### Alkaloids

#### Corynoxine isomers

**Fig. 5:**
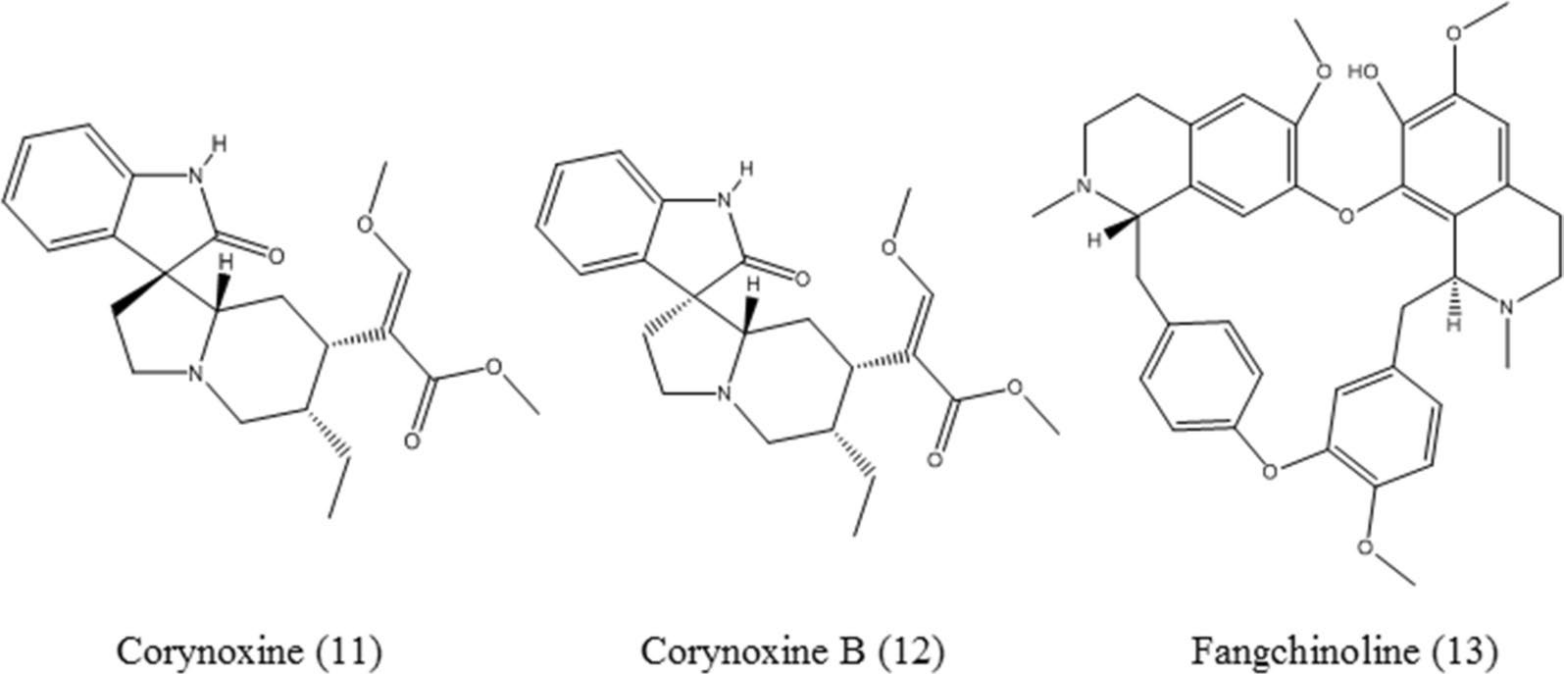
Chemical structures of alkaloids, including corynoxine, corynoxine B and fangchinoline

Corynoxine (**11**–**12**, Fig. 5) is isolated from *Uncaria rhynchophylla* (Miq.) Miq.ex Havil. and induces the nuclear translocation of TFEB. Corynoxine and corynoxine B decrease Aβ through increasing the degradation of APP and CTF, promoting autophagy and lysosome biogenesis [63].

#### Fangchinoline

Fangchinoline (**13**, Fig. 5) is an alkaloid isolated from *Stephania tetrandra* S. Moore. Fangchinoline can promote the nuclear translocation of TFEB and the expression of its target genes at an early stage of autophagy in non-small cell lung cancer (NSCLC) cells. However, in the late period of treatment, fangchinoline inhibits the fusion of the autophagosome and lysosome, and affects lysosomal function, leading to a decrease in the autophagic flux [64]. Accordingly, whether a compound induces or inhibits autophagy is a complex problem, and it depends on the treatment time. It is necessary to screen autophagy inducers or inhibitors by using a real-time monitored method.

### Terpenoids

#### Pseudoginsenoside-F11

**Fig. 6:**
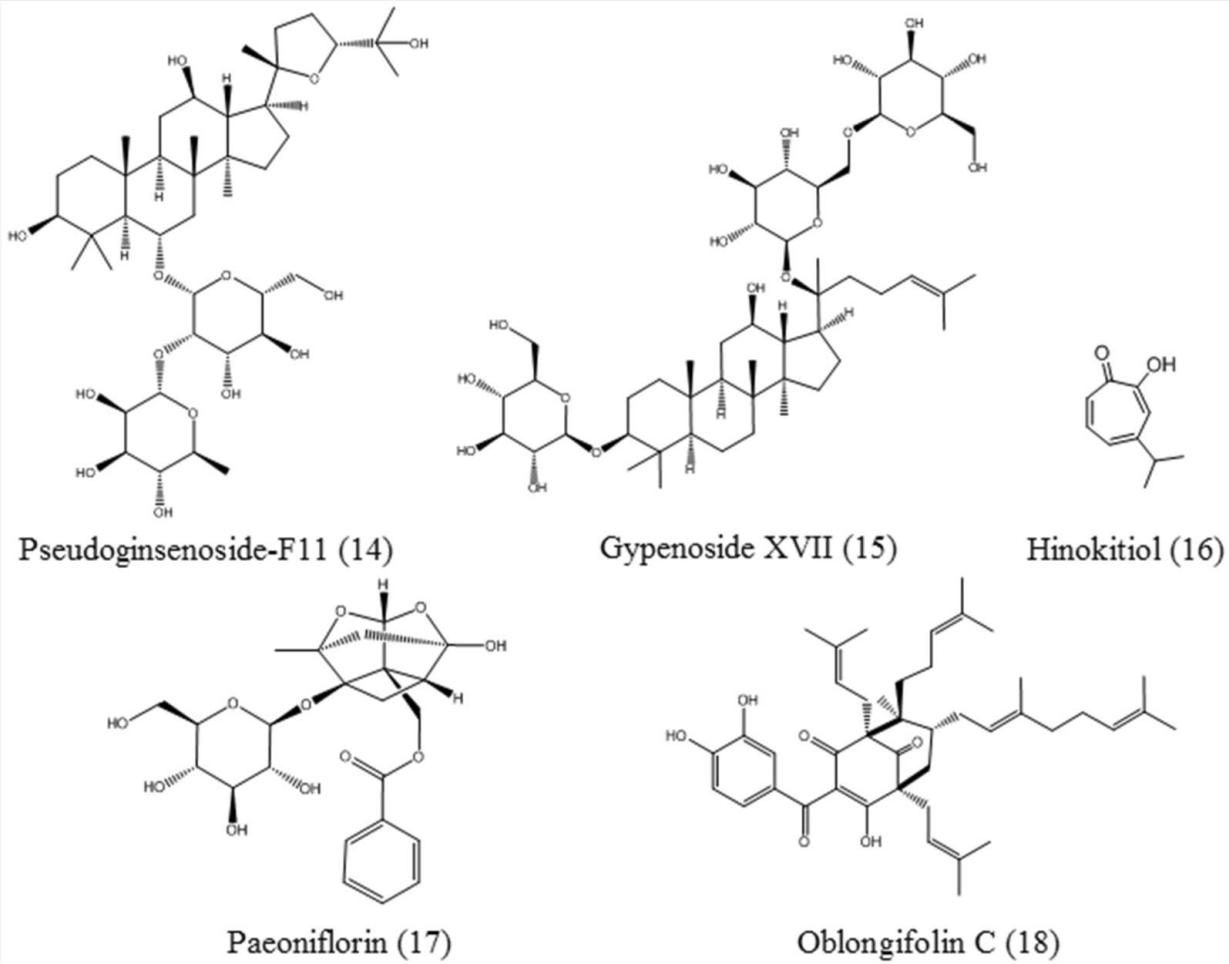
Chemical structures of terpenoids, including pseudoginsenoside-F11, gypenoside XVII, hinokitiol, paeoniflorin, and oblongifolin C

Pseudoginsenoside-F11 (**14**,Figure 6), an ocotillol-type saponin that is derived from leaves of *Panax pseudoginseng subsp. himalaicus* HARA (Himalayan Panax), has beneficial effects in vitro and in vivo on central nervous system disorders, such as PD and AD [87, 88]. Pseudoginsenoside-F11 facilitates the nuclear translocation of TFEB through mTOR inhibition. Pseudoginsenoside-F11 has been proven to increase the uptake and degradation of oligomeric Aβ in microglia, most likely by promoting the maturation of endosomes and improving the function of the lysosome [65].

#### Gypenoside XVII

Gypenoside XVII (**15**, Fig. 6), a major saponin abundant in ginseng and *Panax notoginseng*, has been shown to rescue autophagy flux and enhance lysosome biogenesis through TFEB activation. Gypenoside XVII facilitates the autophagic removal of Aβ in cellular and rodent models of AD. Meanwhile, gypenoside XVII restores the spatial learning and memory of APP/PS1 mice [66].

#### Hinokitiol

Hinokitiol (β-thujaplicin) (**16**, Fig. 6), a monoterpenoid compound extracted from the wood of cupressaceous, exhibits multiple bioactivities such as anti-inflammatory, anti-bacterial and anticancer activities through apoptosis and autophagy. Hinokitiol activates the TFEB nuclear translocation for autophagy and lysosomal biogenesis, and induces cancer cell death [67].

#### Paeoniflorin

Paeoniflorin (**17**, Fig. 6), which is the major bioactive substance of *Moutan cortex* and *Paeonia lactiflora* Pall., has been reported to have various functions, including anti-oxidation, anti-inflammation and neuroprotection [89]. Paeoniflorin upregulates TFEB in a nuclear factor-YA (NF-YA)-dependent manner to activate autophagy, inhibit aggregation of mutant AR and to ameliorate motor phenotypes in SBMA cells and transgenic animal models [37].

#### Oblongifolin C

Oblongifolin C (**18**, Fig. 6) is a natural compound from the *Garcinia yunnanensis* Hu. Oblongifolin C can induce TFEB dephosphorylation and subsequent nuclear translocation by inhibiting mTORC1. Oblongifolin C improves autophagosome maturation but blocks autophagosome–lysosome fusion by engaging the SNARE protein syntaxin 17 (STX17). In addition, the combination of oblongifolin C and hydroxycitrate can further suppress HeLa cells growth [68].

### Steroids

#### Ouabain

**Fig. 7:**
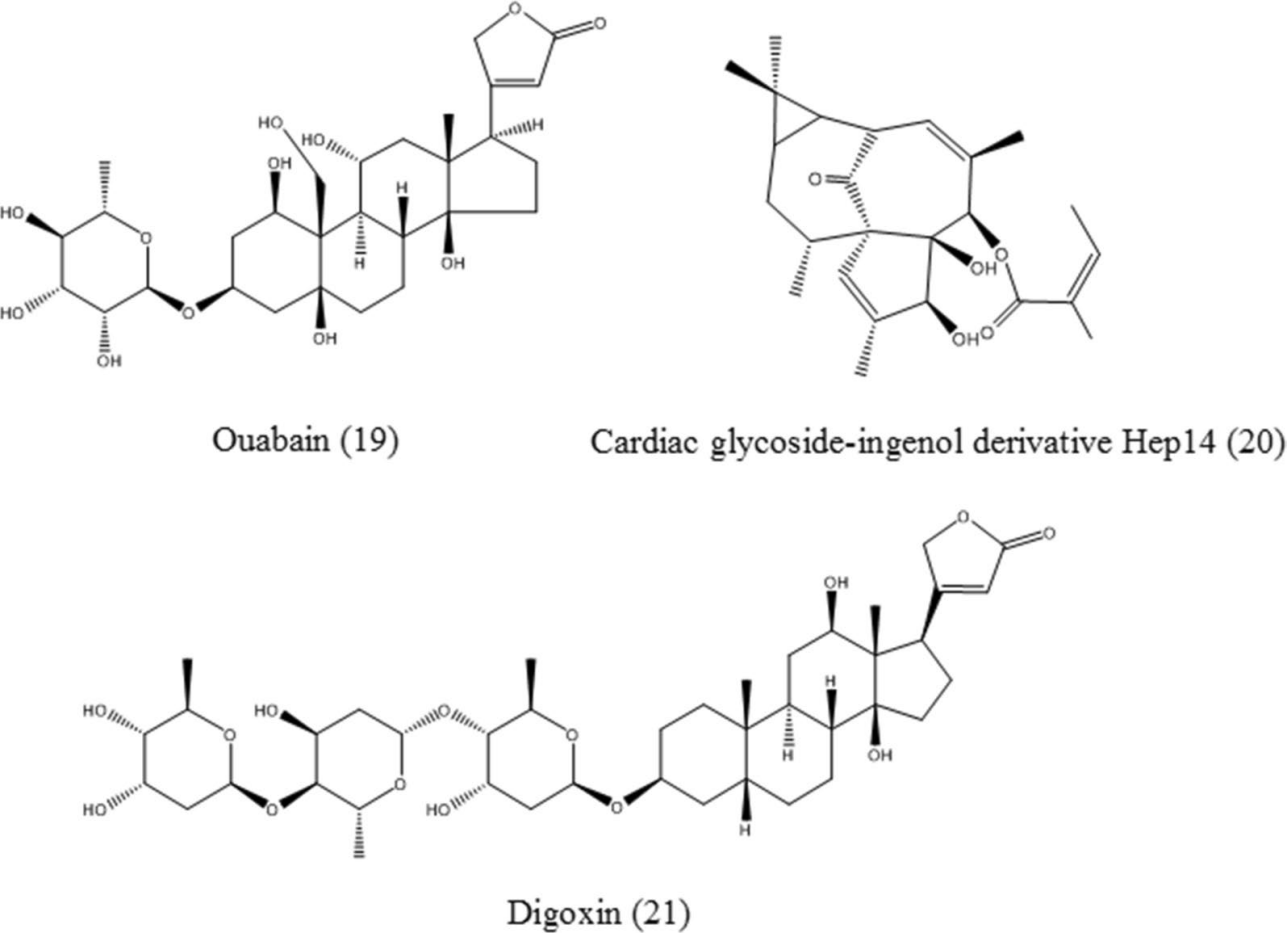
Chemical structures of steroids, including ouabain, cardiac glycoside-ingenol derivative Hep14, and digoxin

Ouabain (**19**, Fig. 7), a cardiac glycoside, is an mTOR and Na^+^/K^+^-ATPase inhibitor. It activates TFEB, induces downstream autophagy-lysosomal gene expression, protects against okadaic acid (OA)-induced neuronal damage, reduces p-Tau aggregates, improves the rough-eye phenotype of tau transgenic flies, and restores memory performance in Tau-P301L mice [69].

#### Cardiac glycoside-ingenol derivative Hep14

Cardiac glycoside-ingenol derivative Hep14 (**20**, Fig. 7), derived from *Euphorbia peplus* Linn, activates TFEB through the PKC-GSK3 cascade. In addition, PKC activation further activates JNK2 and p38, which in turn phosphorylate ZKSCAN3. ZKSCAN3 translocates to the cytoplasm, consequently ameliorating transcriptional repression. Thus, HEP14, which controls two protein phosphorylation cascades by PKC to activate lysosomal gene expression, obviously promotes lysosome-dependent clearance of lipid droplets and aggregated proteins in cell models and reduces amyloid β plaques in APP/PS1 mice [70].

#### Digoxin

Digoxin (**21**, Fig. 7) is a natural cardiac glycoside. It has been recently reported that digoxin inhibits Na^+^/K^+^-ATPase, increases the cytosolic Ca^2+^ level, and leads to TFEB dephosphorylation and activation. TFEB activation induced by digoxin engages lipid catabolism and ameliorates metabolic syndromes [71].

### Disaccharides

#### Trehalose

**Fig. 8:**
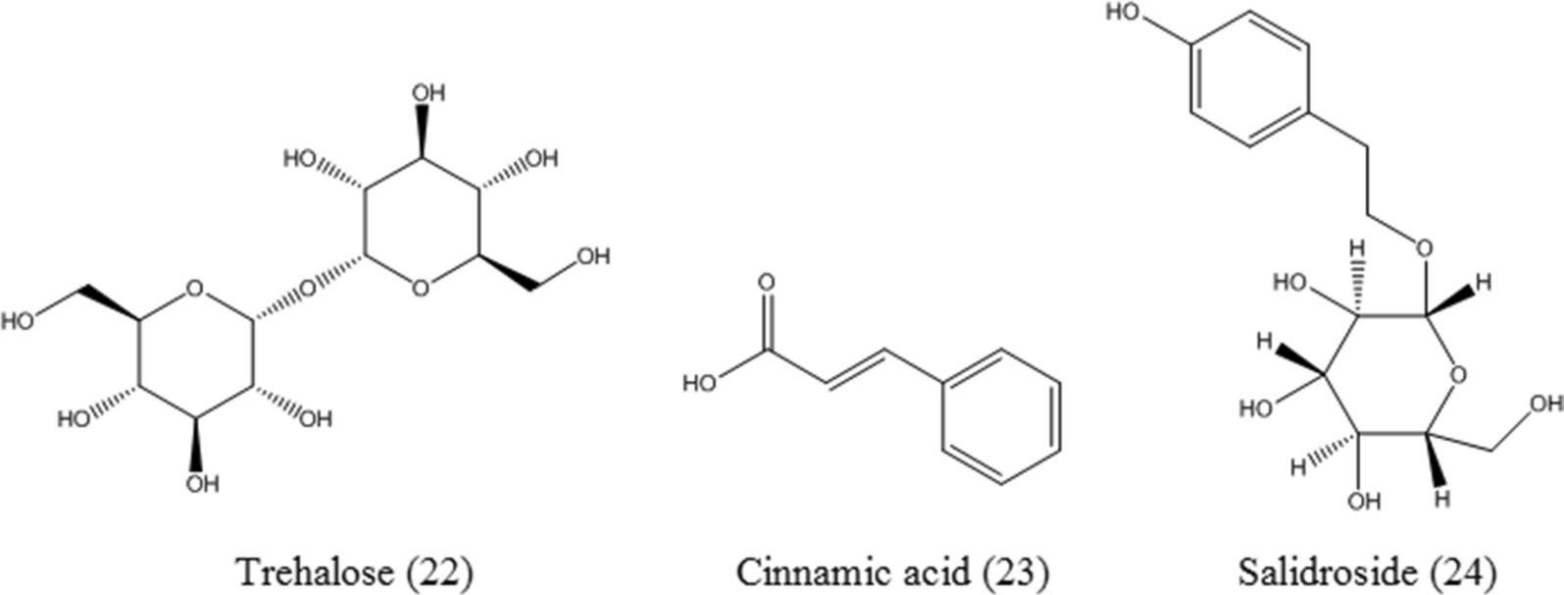
The chemical structures of trehalose, cinnamic acid, and salidroside

Trehalose (**22**, Fig. 8), a disaccharide composed of two glucose molecules, can be synthesized by many fungi and plants. Activation of TFEB by trehalose in an mTOR-independent manner enhances autophagy and the clearance of TDP-43 aggregates, which are related to ALS diseases [38]. Trehalose has been demonstrated to induce TFEB nuclear translocation and upregulate TFEB target genes. TFEB silencing offsets the degradation of misfolded protein in ALS and SBMA motoneuron diseases. Notably, melibiose and lactulose exert similar effects [72]. Trehalose activates TFEB via Akt inhibition and enhances cellular clearance to reduce neuropathology in Batten disease [73]. Trehalose induces TFEB, driving the macrophage autophagy-lysosome system to reduce atherosclerotic plaque burden [90].

### Others

#### Cinnamic acid

Cinnamic acid (**23**, Fig. 8), an aromatic carboxylic acid, is abundant in vegetables, fruits, and grains. It has been demonstrated that cinnamic acid activates PGC-1α to upregulate TFEB and it induces lysosomal biogenesis in primary brain cells. Moreover, cinnamic acid treatment can reduce the amyloid plaque burden and improve the memory and behavioral performance of 5×úFAD mice in a PGC-1α-dependent manner [74].

#### Salidroside

Salidroside (**24**, Fig. 8), a phenylpropanoid glycoside isolated from the plant *Rhodiola rosea* L., has been proven to possess anti-inflammatory, anti-oxidant, and anticancer properties [91–93]. Salidroside activates TFEB nuclear translocation and increases TFEB reporter activity, which contributes to lysosomal biogenesis and the expression of autophagy-related genes. Salidroside promotes apoptosis and induces autophagy by targeting the ROS-TFEB signaling pathway in human chondrosarcoma cells. Additionally, inhibition of autophagy may improve the antitumor activity of salidroside [75].

## Discussion

ALP, as a vital cellular degradative machinery, is involved in a variety of human diseases. TFEB, coordinating autophagy induction with lysosomal biogenesis, makes it an attractive therapeutic target. Thus far, it has been reported that some nonnatural products can activate TFEB, including rapamycin, fubendazole [94], alexidine dihydrochloride [71], ezetimibe [95], gemfibrozil [96], aspirin [97] and so on. Compared with nonnatural products, natural products have played a significant role in health maintenance and disease control due to their properties of fewer side effects and multitargeted activity. In addition, natural products have been used as lead compounds for structural modification to synthesize derivatives, such as LY294002, a commonly used kinase inhibitor [98]. However, currently known TFEB activators from natural products are partial mTOR inhibitors. The mTOR kinase participates in the regulation of cell growth and metabolism, and thus mTOR inhibitors are likely to elicit undesirable side effects leading to limitations of their long-term use [99].

TFEB overexpression is related to the progression of cancers. At the same time, inefficient activation of TFEB may not only lead to ineffective treatment but also aggravate disease [2]. How to balance the beneficial and harmful effects of autophagy? Given the complexity of the ALP machinery, TFEB activation needs to be tightly regulated. Some compounds are autophagy inducers or inhibitors at specific time points. Whether these autophagy inducers or inhibitors have the opposite effect at other time points? For example, fangchinoline promotes the nuclear translocation of TFEB and expression of its target genes at an early stage of autophagy. But in the late period of treatment, fangchinoline inhibits the fusion of the autophagosome and lysosome, and affects lysosomal function, leading to a decrease in the autophagic flux [64]. Accordingly, it is necessary to screen autophagy inducers or inhibitors by using a real-time monitored method. In addition, the concentration to effectively activate TFEB may be relatively higher than the reported blood concentration seen in human clinical experiments, such as quercetin. The dose and duration will have to be further discussed for the treatment of human diseases.

Although there have been no TFEB activators used in the clinic so far, some drugs already in use have effects on TFEB activation. Whether their pharmacological effects are partly related to TFEB activation is worthy of further study. Taken together, caution should be applied during the use of TFEB activators for disease treatment.

## Conclusions

TFEB is an attractive target due to its engagement in multiple human diseases associated with ALP dysfunction. TCM-derived natural products have been valued as important sources for drug discovery because of their well-documented therapeutic efficacies. Although some natural products have been reported to activate TFEB and ameliorate phenotypes, their molecular properties, the complex machinery, and the lack of clinical experimental evidence limits their further development. Furthermore, the discovery of clinically available TFEB activators may be a promising strategy for the treatment of diseases associated with ALP dysfunction.

## Data Availability

Data sharing is not applicable to this article as no datasets were generated or analyzed during the current study.

## Abbreviations

ALP: Autophagy-lysosome pathway
TFEB: Transcription factor EB
TCM: Traditional Chinese medicine
UPS: ubiquitin-proteasome system
CMA: Chaperone-mediated autophagy
PI3K: Class III phosphatidylinositol 3-kinase
CLEAR: Coordinated lysosomal expression and regulation
mTOR: Mammalian (or Mechanistic) target of rapamycin
mTORC: mTOR complex
Raptor: Regulatory associated protein of mTOR
GβL: G protein β-subunit-like protein
PRAS40: Proline-rich Akt substrate of 40 kDa
DEPTOR: DEP domain-containing mTOR-interacting protein
AMPK: AMP-activated protein kinase
Rictor: Rapamycin-insensitive companion of mTOR
SIN: Stress-activated protein kinase-interacting protein
PROTOR: Protein observed with Rictor
ERK2/MAPK1: Extracellular signal-regulated kinase 2
MCOLN1: Calcium channel mucolipin 1
PGC-1α/PPARGC1A: Peroxisome proliferator activated receptor-γ coactivator 1α
LSDs: Lysosomal storage disorders
GAA: Acid alpha-glucosidase
GC: Glucocerebrosidase
MSD: Multiple sulfatase deficiency
GAGs: Glysosaminoglycans
MPS: Mucopolysaccharidosis
HD: Huntington’s disease
HTT: Huntingtin
polyQ: Polyglutamine
PD: Parkinson’s disease
AD: Alzheimer’s disease
Aβ: β-Amyloid peptides
NFTs: Neurofibrillary tangles
APP: Amyloid precursor protein
PS: Presenilin
SBMA: Spinal and bulbar muscular atrophy
AR: Androgen receptor
ALS: Amyotrophic lateral sclerosis
TDP-43: Transactive response (TAR) DNA-binding protein 43 kDa
GSK3: Glycogen synthase kinase-3
ROS: Reactive oxygen species
MAO: Monoamine oxidase
H2O2: Hydrogen peroxide
HUVECs: Human umbilical vein endothelial cells
MEFs: Mouse embryonic fibroblasts
CTF-β/α: APP C-terminal fragments
6-OHDA/AA: 6-Hydroxydopamine/ascorbic acid
MPTP: 1-methyl-4-phenyl-1,2,3,6-tetrahydropyridine
NSCLC: Nonsmall cell lung cancer
NF-YA: Nuclear factor-YA
STX17: SNARE protein syntaxin 17
OA: Okadaic acid
